# Evaluation of Chemical Composition, Anticancer, Antioxidant, Antibacterial, and Antidiabetic Activities of *Peucephyllum schottii*

**DOI:** 10.3390/ijms27104497

**Published:** 2026-05-18

**Authors:** Ibrahim M. Aziz, Mohamed A. Farrag, Noura S. Aldosari, Najat A. Y. Marraiki

**Affiliations:** Department of Botany and Microbiology, College of Science, King Saud University, Riyadh 11451, Saudi Arabia; mfarrag@ksu.edu.sa (M.A.F.); nsaldosari@ksu.edu.sa (N.S.A.); najat@ksu.edu.sa (N.A.Y.M.)

**Keywords:** natural products, phytomolecules, GC-MS, anticancer activity, proliferation, apoptosis, free radicals, antibacterial, antidiabetic

## Abstract

*Peucephyllum schottii* is an aromatic desert plant of the family *Asteraceae*, which has little scientific research regarding its phytochemical composition and pharmacological properties. This study aims to evaluate in detail the chemical composition and antioxidant, antibacterial, antidiabetic, and cytotoxic activities of the ethanol extract of *P. schottii* leaves. The chemical composition of the plant extract was analyzed by GC-MS. Total phenolic (TPC) and flavonoid (TFC) contents of the plant were calculated. An antioxidant assay of the plant material was performed by using the DPPH and ABTS tests. The antibacterial activities of *P. schottii* plant material against six pathogenic bacteria were studied by using the agar diffusion and MIC/MBC techniques. Colorimetric analysis, for its part, enabled the assessment of its antihyperglycemic activities (α-amylase and α-glucosidase) and its cytotoxic activities (in MCF-7 and HepG2 cells). The expressions of apoptotic proteins (caspases, Bcl2, and Bax), were analyzed by RT-PCR. The GC-MS findings showed the presence of complex phytoconstituents of *P. schottii* in the form of linoleic acid (19.48%), hexadecanoic acid (15.01%), and vitamin E (12.15%). There is high TPC (118.18 mg of GAE/g) and TFC (75.56 mg of QE/g) in *P. schottii* plant material. The plant showed significant antioxidant (≈105 μg/mL IC_50_ in DPPH and ≈80 μg/mL IC_50_ in ABTS) and broad-spectrum antibacterial activities, mostly against *E. coli* (MIC = 4.68 μg/mL), as well as antihyperglycemic activities against α-amylase (IC_50_ = 334 μg/mL) and α-glucosidase (IC_50_ = 196 μg/mL) enzymes. The plant material showed cytotoxic effects in MCF-7 and HepG2 cells in a concentration-dependent manner (IC_50_ = 78 ± 1.13 μg/mL and 68.23 ± 2.41 μg/mL, respectively). These findings point to *P. schottii* leaf extract’s potential as a natural antioxidant, antibacterial, antidiabetic, and chemopreventive agent.

## 1. Introduction

*Asteraceae*, or the sunflower family, constitutes one of the largest and most successful groups of flowering plants, in excess of 32,000 species, represented in more than 1900 genera [1]. Members of this family of plants can be easily recognized by the characteristic inflorescence in the form of a capitulum, or a collection of tiny florets, which appear to constitute one flower but essentially form an aggregate of numerous minute blooms. Members of the family can literally be found in nearly all terrestrial biomes around the world, with a preponderance in arid, semi-arid, and Mediterranean regions [2].

In addition to its natural preponderance, the *Asteraceae* family has been traditionally used in medicine by many different human cultures throughout history. Many of these species are renowned for their high content of bioactive secondary metabolites. Compounds such as sesquiterpene lactones, flavonoids, polyphenolic acids, polyacetylenes, and essential oils have diverse pharmacological properties [3,4,5]. Members of this family have long been used to treat a variety of ailments, including infections, inflammations, gastrointestinal disorders, and diabetes. Scientific research has shown that plants from the *Artemisia*, *Tanacetum*, *Matricaria*, and *Echinacea* genera showed high antioxidant, antibacterial, anti-inflammatory, and antidiabetic properties [5,6,7,8]. One of the remarkable phytochemicals of that family is Sesquiterpene lactones, which are often reported for their high cytotoxic and anticancer activities [3,9].

Despite the family’s established bioactivity, the chemical and bioactivity profile remains unexplored for most *Asteraceae* species, especially those that are adapted to demanding environments. *P. schottii* (A. Gray), also known as Pygmy Cedar or Desert Fir, is endemic to the arid desert landscapes in the southwestern United States and the northern Mexican territories, where the plants are described as “relatively abundant and widespread” [10]. The tree is evergreen, with considerable stature, and thus poses an enormous gap in scientific knowledge. The tree’s ability to thrive in the arid desert conditions appears to indicate the possibility of it having a particular chemical and bioactivity profile that might be essential to its survival, though this remains unexplored. Research along these lines is not only necessary to develop new knowledge regarding the chemical constitution of the *Asteraceae* family but also provides opportunities to discover new drugs.

Therefore, this study aimed to systematically assess the chemical constituents and bioactivities of *P. schottii* by evaluating its phytochemicals using gas chromatography–mass spectrometry (GC-MS) analysis, measuring the total phenolic content (TPC) and total flavonoid content (TFC), and evaluating its antioxidant, antibacterial, antidiabetic, and cytotoxic activities and the apoptotic mechanism thereof.

## 2. Results

### 2.1. GC-MS Analysis

**Figure 1:**
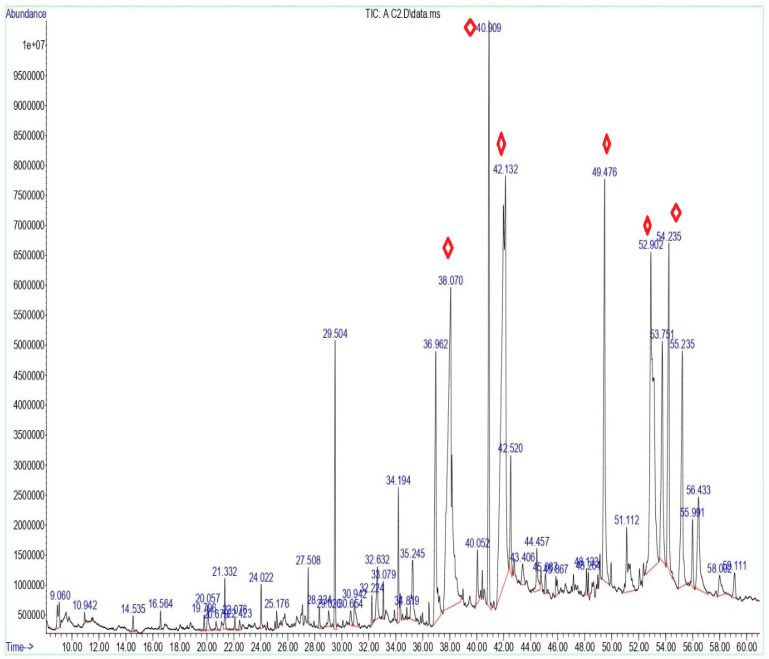
GC-MS chromatograms of the *P. schottii* leaf extract. Every peak in the spectrum corresponds to a known chemical, and a large peak indicates the main component of the extract. The highlighted peaks indicate the major bioactive compounds in the extract.

The GC–MS analysis of the ethanolic extract of *P. schottii* leaves, performed using Wiley and NIST mass spectral libraries with a similarity index greater than 90%, resulted in the identification of 46 compounds, representing diverse chemical classes (Table 1; Figure 1). The GC–MS chromatogram illustrating the detected bioactive constituents is presented in Figure 1, while their corresponding retention times (RTs), peak area percentages, molecular formulas (MFs), and molecular weights (MWs) are summarized in Table 1. The major constituents detected were linoleic acid (19.48%), n-hexadecanoic acid (15.01%), and vitamin E (12.15%), followed by 2-monopalmitin (6.66%), phytol (6.32%), and neointermedeol (2.47%).

### 2.2. TPC and TFC

The ethanolic extract of *P. schottii* leaves was evaluated for TPC and TFC. The extract exhibited a TPC of 118.18 ± 2.93 mg GAE/g dry extract and a TFC of 75.56 ± 1.53 mg QE/g dry extract. These results indicate that the extract is rich in phenolic and flavonoid compounds, suggesting strong potential as a source of antioxidant phytochemicals.

### 2.3. Antioxidant Activity

**Figure 2:**
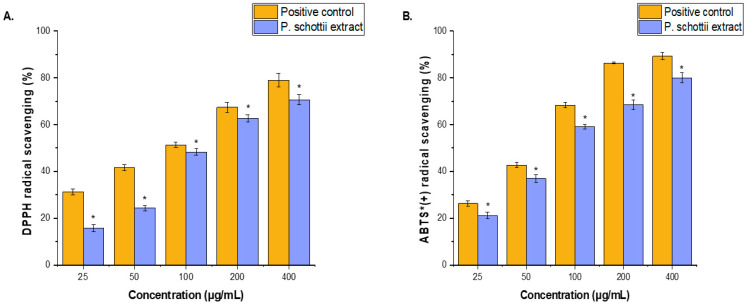
(**A**) The DPPH reducing capacity and (**B**) ABTS^+^ scavenging activity of ethanol extract from the leaves of *P. schottii* at various concentrations (25–400 μg/mL). Ascorbic acid (25–400 μg/mL) served as a positive control. The average value from three independent experiments is shown. The scavenging activity of *P. schottii* extract was significantly lower (*) compared to the positive control at a significance level of *p* < 0.05. ^+^ = radical cation.

The antioxidant activity of the ethanolic extract of *P. schottii* leaves was evaluated using 2,2-diphenyl-1-picrylhydrazyl (DPPH) and 2,2′-azino-bis-(3-ethylbenzothiazoline-6-sulfonic acid) (ABTS) radical scavenging assays, with vitamin C employed as a reference standard (Figure 2A,B). The extract exhibited a clear concentration-dependent increase in radical scavenging activity over the tested range (25–400 μg/mL), reaching 70.64 ± 2.12% and 79.93 ± 2.14% inhibition at 400 μg/mL in the DPPH and ABTS assays, respectively. The IC_50_ values were determined to be ≈105 μg/mL (DPPH) and ≈ 80 μg/mL (ABTS), indicating moderate antioxidant activity. In comparison, vitamin C demonstrated significantly stronger activity, with IC_50_ values of ≈89 μg/mL (DPPH) and ≈64 μg/mL (ABTS). Overall, the extract showed greater efficacy in the ABTS assay than in the DPPH assay, suggesting differential reactivity toward radical species.

### 2.4. Antibacterial Effects of Bioactive Compounds

The antibacterial activity of the ethanolic extract of *P. schottii* leaves was evaluated against six pathogenic bacterial strains using the disk diffusion and broth microdilution methods (Table 2). The extract exhibited broad-spectrum antibacterial activity, with inhibition zones ranging from 10 to 23 mm, depending on the concentration and bacterial strain. The highest activity was observed at 400 μg/mL, particularly against *E. coli* and *P. aeruginosa* (23 mm), followed by *Staphylococcus epidermidis* (21 mm). Overall, Gram-negative bacteria showed slightly higher sensitivity compared to Gram-positive strains.

The determination of minimum inhibitory concentration (MIC) values ranged from 4.68 ± 2.21 to 25 ± 0.00 μg/mL, while the minimum bactericidal concentration (MBC) values ranged from 6.25 ± 0.00 to 50 ± 0.00 μg/mL. Among the tested strains, *E. coli* exhibited the highest susceptibility (MIC = 4.68 ± 2.21 μg/mL), whereas *S. aureus* showed comparatively lower sensitivity (MIC = 25 ± 0.00 μg/mL). These findings demonstrate that the extract possesses both bacteriostatic and bactericidal activities, supporting its potential as a natural antimicrobial agent.

### 2.5. In Vitro α-Amylase and α-Glucosidase Inhibition Activities

**Figure 3:**
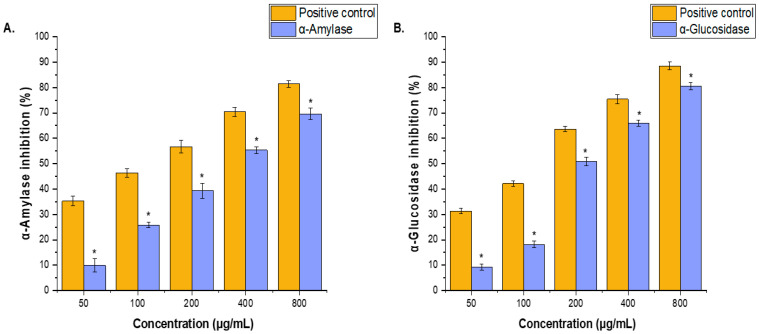
(**A**) α-Amylase and (**B**) α-glucosidase inhibitory activities of ethanol extract from the leaves of *P. schottii* at various concentrations (50–800 μg/mL). Acarbose was used as the positive control (tested at 50–800 μg/mL, consistent with the extract concentration range). The results are the mean values of three replicates ± SD. * *p* < 0.05 vs. positive control.

The inhibitory activity of the ethanolic extract of *P. schottii* leaves against α-amylase and α-glucosidase enzymes was evaluated in vitro, and IC_50_ values were determined (Figure 3A,B). The extract exhibited concentration-dependent inhibitory effects against both enzymes over the tested concentration range (50–800 μg/mL) compared with the positive control. In the α-amylase assay, the extract showed inhibition values ranging from 9.84% to 69.64%, with an estimated IC_50_ value of approximately 334 μg/mL, whereas the positive control exhibited stronger activity with an IC_50_ value of approximately 136 μg/mL. Similarly, in the α-glucosidase assay, the extract demonstrated inhibition percentages ranging from 9.19% to 80.47%, with an estimated IC_50_ value of approximately 196 μg/mL, compared with 136 μg/mL for the positive control. These findings indicate that the extract possessed greater inhibitory activity against α-glucosidase than α-amylase, suggesting promising antidiabetic potential through the inhibition of carbohydrate-hydrolyzing enzymes.

### 2.6. Cell Cytotoxicity and Apoptosis Markers

**Figure 4:**
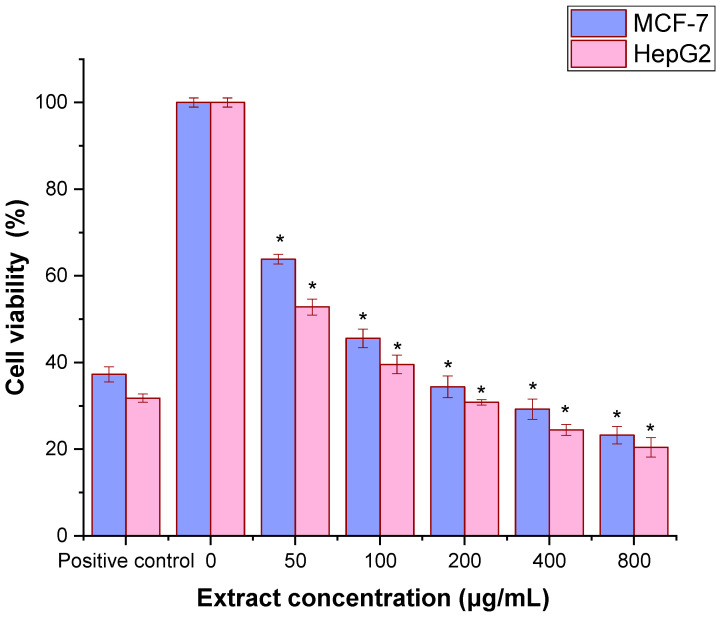
The effect of ethanol extract of the leaves of *P. schottii* on cell viability. The cells were exposed to the specified concentration (0–400 μg/mL) of leaf extract for 24 h, and the viability of the cells was determined by using the MTT assay. Cell viabilities are shown as percentages, and the untreated cells were regarded as 100% viable. The mean ± SD is presented from three independent experiments (* *p* < 0.05) compared to non-treated cells (negative control).

The cytotoxic effects of the ethanolic extract of *P. schottii* leaves were evaluated against MCF-7 and HepG2 cell lines using the thiazolyl blue tetrazolium bromide (MTT) assay (Figure 4). The extract exhibited dose-dependent inhibition of cell proliferation in both cell lines when compared to the standard drug cisplatin. The calculated IC_50_ values were 78 ± 1.13 μg/mL for MCF-7 cells and 68.23 ± 2.41 μg/mL for HepG2 cells, indicating a higher sensitivity of HepG2 cells to the extract. A significant reduction in cell viability was observed at higher concentrations (*p* < 0.05).

**Figure 5:**
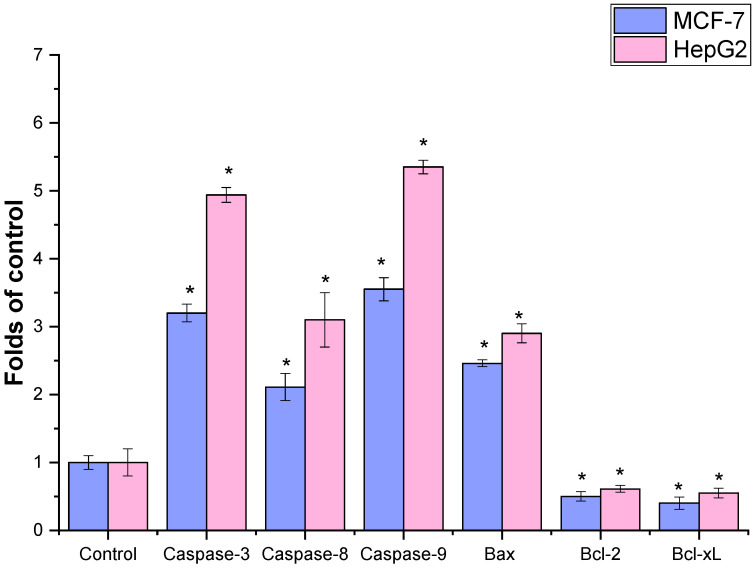
The effect of ethanol extract of the leaves of *P. schottii* on the induction of apoptosis in human MCF-7 and HepG2 cells. The cells were treated without or with IC_50_ values of (88 μg/mL for MCF-7 and 75 μg/mL for HepG2) ethanolic leaves of *P. schottii* extract for 48 h; RNA was collected, isolated, and subjected to rRT-PCR. Ethanol extracts from leaves significantly stimulate the activity of a percentage of the apoptotic population (caspase-3, 8, 9, and Bax) and anti-apoptotic genes (Bcl-xL and Bcl-2) compared to the control cells. The results are represented as the mean ± SD of three independent experiments (* *p* < 0.05 compared to non-treated cells (control)).

To further elucidate the mechanism of cytotoxicity, the expression of apoptosis-related genes was analyzed using real-time reverse transcription polymerase chain reaction (rRT-PCR) (Figure 5). Treatment with IC_50_ concentrations of the extract resulted in a significant upregulation of pro-apoptotic genes, including caspase-3, caspase-8, caspase-9, and Bax (*p* < 0.01), in both MCF-7 and HepG2 cells. Conversely, the expression levels of anti-apoptotic genes (Bcl-2 and Bcl-xL) were significantly downregulated compared to untreated control cells (*p* < 0.05). These findings suggest that the ethanolic extract of *P. schottii* induces apoptosis through activation of both intrinsic and extrinsic apoptotic pathways, thereby contributing to its cytotoxic effects.

## 3. Discussion

The chemical composition of the *P. schottii* leaf extract has been analyzed. The data obtained help in understanding the various bioactivities and chemotaxonomic relationships that the plant might exhibit. A moderate extraction yield with a diverse phytochemical composition mainly comprising fatty acids, lipids, and terpenoids was observed. For the extraction yield, about 23.2% of the total quantity of dried herbal material extracted gave the desired composition. This demonstrates that the extraction procedure adopted was efficient in the extraction of the polar and nonpolar solubilized phytochemicals. Although the extraction yield percentage of *P. schottii* cannot be directly compared with values presented in other literature works focusing on other various plant materials, this value likely falls within the moderate to high range described for other leaf extracts from several drought-adapted plants using similar solvents, implying the existence of a substantial quantity of phytochemicals that would potentially be extracted. Refs. [5,11] found that extracting essential oils from *Asteraceae* plants yields approximately 0.29%.

GC-MS analysis of phytochemicals revealed 46 compounds with a very diverse phytochemical composition, mostly consisting of lipids and terpenoids. Linoleic acid (19.48%) and n-hexadecanoic acid (palmitic acid, 15.01%) were the most common compounds, accounting for more than one-third of the overall content analysis. Other ingredients included vitamin E or tocopherols (12.15%), glycerol lipids, such as 2-monopalmitin (6.66%), the acyclic diterpenoid phytol (6.32%), and fatty acid molecules like Oleamide (4.8%). These molecules, notably linoleic and palmitic acids, act as foundation chemicals for lipids and membranes. The plant extract’s anti-inflammatory and antibacterial properties could increase its total bioactivity [12,13,14]. Vitamin E or α-tocopherol, which has a great liposoluble antioxidant property, plays a vital function in defending plant architectures against oxidative stress and has great value and benefit as nutrition and medication [15]. The high presence of this vitamin in *P. schottii* might, therefore, establish the plant as a great potential source.

Despite the limited phytochemical information available for *P. schottii*, the occurrence of sesquiterpenoids (e.g., caryophyllene and α-santonin) and diterpenoids (including phytol and neophytadiene) indicates a potential chemotaxonomic association with the *Asteraceae* family. Many *Asteraceae* species have a large presence of terpenoids, especially sesquiterpene lactones [9], which are generally known for imparting their characteristic bitterness and their biological properties like anti-inflammatory, antimicrobial, and cytotoxic effects [3]. The presence of α-santonin, a well-studied sesquiterpene lactone with recognized antiparasitic, anticancer, anti-inflammatory, and immunomodulatory activities, confirms the resemblance and suggests distinct biological features for *P. schottii* [16]. Phytol, a D-terpene-alcohol derivative of chlorophyll, is commonly found in green plant extracts and has been linked to antibacterial, anti-inflammatory, and cosmeceutical effects [17,18,19]. The GC-MS analysis of essential oil extracts from *Matricaria chamomilla* (*Asteraceae*) found a comparable composition for sesquiterpenoids such as caryophyllene and trans-bisabolene epoxide [5]. The GC-MS analysis of the methanolic extracts of *Artemisia judaica* (*Asteraceae*) revealed some similarity in composition with *P. schottii,* which contains oxygenated hydrocarbons (vitamin D and Ethyl iso-allocholate), fatty acids (Heptadecanoic acid), and Terpenes (α-santonin) [20]. Occasionally, the chemical composition of *Artemisia campestris* (*Asteraceae*) revealed similar weightages of phytol (6.6%), whereas caryophyllene oxide revealed relatively higher weightages (5.8%) [8]. The chemical profile, which includes fatty acids, terpenoids, and tocopherol compounds, points toward the metabolic profile of *P. schottii*, which is adapted to survive in arid climatic conditions and might impart properties similar to those of other species. Furthermore, GC-MS analysis found α-santonin and other sesquiterpenoids in *P. schottii*, suggesting its chemotaxonomic association with the *Asteraceae* family, which is consistent with previous phytochemical studies that found sesquiterpene lactones [10].

Polar and heat-labile metabolites, including many phenolic glycosides, flavonoids, and organic acids, usually require derivatization procedures (such as methylation) to increase their volatility and thermal stability before GC-MS analysis [11]. For this reason, of the 46 components identified in our study, the majority are lipophilic compounds like fatty acids, terpenoids, sterols, and tocopherol. Accordingly, the phytochemical profile presented herein should be interpreted as a preliminary characterization of the extract rather than a comprehensive representation of its complete metabolomic composition.

*P. schottii* leaf ethanol extract was rich in phenolic compounds and flavonoids, with concentrations of 118.18 mg GAE/g and 75.56 mg QE/g extract, respectively. The large TPC content of the extract indicates that the extract contains abundant hydrogen donors that can scavenge the free radicals. The latter aspect is of prime importance given the fact that flavonoids are one of the major classes of phenolics and are well documented for their wide spectrum of bioactivities, like antioxidant, anti-inflammatory, and antibacterial properties [21]. The measured TPC of the extract (118.18 mg GAE/g) was far greater than many earlier reported values for leaf extracts of many other plant species growing under arid and semi-arid conditions. This high phenolic production may be an adaptive trait that helps the plant thrive under an arid environment, where such compounds protect the plant from UV-mediated oxidative injury [22]. Many members of the family of *Asteraceae* are well known for their TPC. Leaf extracts of several wild *Asteraceae* plants native to the Mediterranean regions showed lower TPC values ranging from 6.5 to 13.3 g kg^−1^ DM, depending on plant type and solvent used, thereby placing *P. schottii* well within the competitive range [23]. The value of the TFC of the extract, at 75.56 mg QE/g, is also noteworthy. Many earlier studies on flavonoid-bearing members of the *Asteraceae* family, like *Calendula officinalis* or *Echinacea purpurea,* showed values for the latter parameter in the range of TFC = 101–147 mg QE/100 g extract, thereby establishing *P. schottii* as an extract rich in the compound [7,24]. The large TPC and TFC abundantly establish the fact that the *P. schottii* extract contains good in-built antioxidant properties.

The ethanolic extract of *P. schottii* leaves exhibited strong antioxidant activity, with IC_50_ values of ≈105 μg/mL in the DPPH assay and ≈80 μg/mL in the ABTS assay. These results indicate a high capacity to donate hydrogen atoms or electrons, thereby stabilizing free radicals and interrupting radical chain reactions. The lower IC_50_ value observed in the ABTS assay suggests comparatively higher scavenging efficiency in this system. Overall, the concentration-dependent activity confirms the potent antioxidant potential of the extract. However, direct comparison with literature values expressed as μM Trolox equivalents per gram of dry weight is not appropriate due to differences in units, reference standards, and assay conditions. Nevertheless, when compared with other studies reporting IC_50_ values of plant extracts in μg/mL under similar experimental conditions, the antioxidant activity of *P. schottii* extract is highly competitive or superior to several Asteraceae species, such as *Taraxacum officinale*, *Tanacetum vulgare*, *Chrysanthemum morifolium*, and *Ageratum conyzoides* [6,25]. The remarkable activity can easily be attributed to the previously reported data on the respective phytochemistry of the plants. The large amounts of an antioxidant agent, vitamin E, can easily explain the present findings [26]. Vitamin E has already been recognized for antimicrobial activities in lipid environments, and the large amounts observed can easily contribute to the remarkable activity of the sample in the ABTS test [27]. It also exerts synergetic effects, enabling potential combination with other compounds, like linoleic acid and terpenoids, resulting in added antioxidant effects.

The ethanolic extract of *P. schottii* had high antibacterial activity against both Gram-positive and Gram-negative pathogens, with low MIC and MBC values, indicating a bactericidal mechanism of action. The strong efficacy against *E. coli* (MIC 4.68 µg/mL) is highly significant. This effectiveness across bacterial cell wall types suggests that the extract contains compounds that can modify core microbial structures or functions. This antibacterial activity is much higher than that of various plant extracts from the *Asteraceae* family. As an example, the antimicrobial effects of some essential oils of *Centaurea* species ranged from 500 to 1000 µg/mL against *E. coli*, 31–125 µg/mL against *S. aureus* [28], 20 μg/mL against *K. pneumoniae* and *P. aeruginosa* [29], and 32 μg/mL against *E. faecalis* [30]. Also, the species of *Baccharis* has weak antibacterial activity against *E. coli* (1000 μg/mL), *S. aureus* (>2000 μg/mL) [31], *K. pneumoniae* (52.5 μg/mL), and *P. aeruginosa* (13.125 μg/mL) [32]. The broad-spectrum effect of the ethanolic extract of *P. schottii* could be attributed to its rich phytochemical composition. For example, n-hexadecanoic acid (palmitic acid) breaks bacterial cell membranes [33], phytol has antibacterial properties [34], and α-santonin has been used as an antimicrobial agent [35]. Furthermore, high levels of flavonoids and other phenolics can inhibit bacterial enzymes and reduce membrane integrity [4]. This multi-component attack most likely reduces the development of bacterial resistance, which increases the extract’s potential as a source of new antibacterial leads or synergists.

The extract showed slightly stronger antibacterial activity against Gram-negative bacteria, especially *E. coli* and *P. aeruginosa*, than against Gram-positive strains, despite the common belief that Gram-negative bacteria are more resistant because of their lipopolysaccharide-rich outer membrane. Similar findings have been reported previously, suggesting that antibacterial activity is not determined solely by bacterial cell wall structure [36]. The enhanced activity may be related to the high levels of bioactive compounds identified in *P. schottii*, including linoleic acid, *n*-hexadecanoic acid, phytol, and α-santonin. These compounds can disrupt bacterial membranes, increase membrane permeability, and interfere with essential cellular processes [28,29,30,31,32,33,34,35]. In addition, some Gram-positive bacteria may possess intrinsic resistance mechanisms, such as efflux pumps, that reduce susceptibility to phytochemicals. Similar preferential activity against Gram-negative bacteria has also been reported for other plant extracts rich in fatty acids and terpenoids [28,29,30].

The extract had significant antidiabetic efficacy against α-amylase (334 µg/mL) and α-glucosidase (196 µg/mL). These IC_50_ values are comparable to those of many researched antidiabetic herbs. Extracts from well-known species, such as *Syzygium cumini*, typically have similar or greater IC_50_ values [37]. Several studies reported the historical antidiabetic activities of *Asteraceae* plants in folk medicine, such as *Achillea asiatica*, *Eclipta prostrata*, *Cynara cardunculus* L., and *Achyrocline alata* (Kunth) DC [38]. Recent studies on *Asteraceae* plants induced α-amylase inhibition by *Cynara scolymus* (IC_50_ = 72.22 μg/mL) [39], *Eclipta prostrata* L. (IC_50_ = 322 μg/mL) [40], and *Solidago virgaurea* L. [41]. The high flavonoid and phenolic content, as well as certain secondary metabolites, such as terpenoids, are linked to this activity, as these compounds can inhibit α-amylase and α-glucosidase enzymes through hydrogen bonding and hydrophobic interactions [42,43,44]. This inhibitory property makes *P. schottii* a good candidate for further development as a functional food or additional medication for type 2 diabetes management.

Finally, the extract exhibited dose-dependent and selective cytotoxicity against MCF-7 (breast) and HepG2 (liver) cancer cells, with IC_50_ values of 78 ± 1.13 µg/mL and 68.23 ± 2.41 µg/mL, respectively. More importantly, the mechanistic study confirmed the activation of both the intrinsic (mitochondrial) and extrinsic (death receptor) apoptotic pathways, as evidenced by the upregulation of caspase-9, caspase-8, and caspase-3, as well as the pro-apoptotic protein Bax, and the downregulation of the anti-apoptotic proteins Bcl-2 and Bcl-XL. The plants of the *Asteraceae* family are rich in sesquiterpenoid lactones, which are proven anticancer agents [45]. For example, a previous study reported the cytotoxic activity of *Launaea procumbens* (Roxb.) against leukemia (K562), cervix (HeLa), pancreatic (MIA-Pa-Ca-2), and breast (MCF-7) cell lines, linked to its composition of phenolic and terpenoid phytochemicals [46]. *Achillea millefolium* and *C. officinalis* showed antitumor potential against human pancreatic cancer cells MIA PaCa-2 and PANC-1 by inducing early apoptosis and activation of the caspase cascade [47]. Also, *Euryops floribundus* showed antiproliferative activities against prostate (DU-145 and PC-3) and uterine leiomyosarcoma (SK-UT-1) cancer cell lines [48]. Several compounds in *P. schottii* are known apoptosis inducers: vitamin E (tocopherol) derivatives have shown pro-apoptotic effects in cancer cells [49]; linoleic acid and its derivatives can modulate apoptotic signaling [12], and sesquiterpene lactones like α-santonin are renowned for their cytotoxic activities, often involving ROS generation and mitochondrial dysfunction [3]. The synergistic interaction of various chemicals within the crude extract is most likely responsible for its powerful and mechanistically defined anticancer activity, which calls for *in vivo* validation and compound isolation studies.

The current work is the first to assess *P. schottii*’s phytochemistry and bioactivity, revealing its novel promise as a source of multi-target therapeutic chemicals. The main innovation is the simultaneous identification of a rich chemical profile—particularly high concentrations of vitamin E, linoleic acid, and various terpenoids—as well as the demonstration of potent, broad-spectrum antibacterial, antidiabetic, and pro-apoptotic anticancer activities in a single extract. Although GC–MS analysis coupled with NIST/Wiley library matching (similarity index > 90%) enabled reliable preliminary identification of phytoconstituents, definitive structural confirmation and precise quantification would require more advanced analytical approaches, such as high-resolution mass spectrometry or liquid chromatography–mass spectrometry (LC–MS/HPLC–MS), in future investigations. Furthermore, the GC–MS method employed in this study is inherently limited to the detection of volatile and semi-volatile constituents of the ethanolic extract. As a result, non-volatile, polar, and thermolabile metabolites may be underrepresented or undetected without prior derivatization or the use of complementary analytical platforms. Accordingly, the phytochemical profile presented herein should be interpreted as a preliminary characterization of the extract rather than a comprehensive representation of its complete metabolomic composition. However, the study is limited to in vitro models, and the identified activities stem from the crude extract. The bioactive molecules causing each effect have yet to be identified and described. A notable limitation of the current study is the absence of cytotoxicity evaluation on normal (non-cancerous) human cell lines. Consequently, the Selectivity Index (SI) could not be determined. The SI, calculated as the ratio of the IC_50_ value in normal cells to that in cancer cells, is an important parameter for assessing the therapeutic potential and safety profile of candidate anticancer agents. In the present study, cytotoxic activity was evaluated only against cancer cell lines (MCF-7 and HepG2), which limits assessment of the extract’s preferential toxicity toward malignant cells. Therefore, future studies should include normal human cell lines, such as HEK-293, HUVEC, human dermal fibroblasts, or primary hepatocytes, to evaluate selective cytotoxicity, determine the therapeutic window, and better establish the safety profile of the *P. schottii* extract before progressing to *in vivo* efficacy and toxicity investigations. In addition, the absence of experimentally determined MIC and MBC values for ciprofloxacin is acknowledged as a limitation; therefore, future research will include the determination of MIC/MBC values for ciprofloxacin under standardized conditions, alongside comparative evaluation with the plant extracts to enable a more rigorous assessment of antimicrobial potency and potential synergistic effects. The bioactive molecules responsible for each observed effect also remain to be isolated and characterized.

## 4. Materials and Methods

### 4.1. Preparation of Plant Extract

Leaves of *P. schottii* were collected from Ibb City, Yemen, and identified by Prof. Dr. Mohammed Fasil (Department of Botany and Microbiology, College of Science, King Saud University). A voucher specimen (KSU No. 178560) was deposited in the University herbarium. The leaves were washed with distilled water, air-dried at room temperature for 3 weeks, and ground into a fine powder.

Extraction was performed using 80% ethanol following a modified method of Sak et al. (2017) [50]. Briefly, 50 g of leaf powder was macerated in cold maceration in 100 mL of aqueous ethanol (80%) for 48 h at room temperature with intermittent shaking. The mixture was filtered (Whatman No. 1), and the filtrate was concentrated at 40 °C using a rotary evaporator. The crude extract was stored at −20 °C until use. Extraction yield was expressed as % (*w*/*w*) dry weight (g extract/100 g dried material).

### 4.2. Determination of Phytochemicals in Leaf Extract

The chemical composition of ethanolic extracts of *P. schottii* leaves was analyzed using an Agilent 7890B GC–MS system (Agilent Technologies, Santa Clara, CA, USA). Prior to analysis, samples were diluted with acetone at a ratio of 1:9 (*v*/*v*), and 0.9 µL of the diluted extract was injected via an autosampler under split-mode conditions (split ratio 50:1).

Separation of compounds was carried out on a DB-5MS capillary column (30 m length, 0.25 mm internal diameter, and 0.25 µm film thickness) using helium as the carrier gas at a flow rate of 1 mL/min. The oven temperature program ranged from 50 °C to 250 °C, with a total run time of 73 min. Mass spectrometric detection was conducted in full-scan acquisition mode within a mass range of 40–500 m/z, with a scan rate of 1.56 amu/s. The ion source temperature was maintained at 230 °C, and a solvent delay of 2 min was applied. Identification of compounds was achieved by comparing the obtained spectra with reference spectra from the NIST and Wiley libraries. Only compounds with a matching factor exceeding 90% were considered positively identified.

### 4.3. Analysis of TPC and TFC

TPC was determined using the Folin–Ciocalteu method with minor modifications [51]. Briefly, 200 µL of Folin–Ciocalteu reagent (10%) was mixed with 2 mL of deionized water and 100 µL of the sample solution. After incubation, 1 mL of 20% (*w*/*w*) NaHCO_3_ was added, and the mixture was incubated at room temperature for 1 h. Absorbance was measured at 765 nm using a UV–Vis spectrophotometer (Hitachi U2001, Tokyo, Japan). TPC was calculated from a gallic acid calibration curve (y = 0.006x + 0.136, R^2^ = 0.991) and expressed as g gallic acid equivalents (GAE)/g dry weight of extract.

TFC was determined using the aluminum chloride colorimetric method [52] with slight modifications. Briefly, 500 µL of extract (600 µg/mL) was mixed with 0.1 mL of 10% (*w*/*v*) AlCl_3_, 0.1 mL of 1 M potassium acetate, 1.5 mL ethanol, and 2.8 mL distilled water. After incubation at room temperature for 30 min, absorbance was measured at 415 nm using a UV–Vis spectrophotometer (Hitachi U2001, Tokyo, Japan). TFC was calculated using a quercetin calibration curve (y = 0.0015x + 0.112, R^2^ = 0.965) and expressed as mg quercetin equivalents (QE)/g dry weight of extract. All measurements were conducted in triplicate.

### 4.4. DPPH and ABTS Scavenging Assay

The DPPH radical scavenging activity was determined according to Tian et al. (2020) [53] with minor modifications. Briefly, 0.2 mL of ethanol extract at different concentrations (50–400 µg/mL) was mixed with 2 mL of 0.08 mM DPPH solution. The mixture was incubated in the dark for 20 min at room temperature. Absorbance was measured at 517 nm using a UV–Vis spectrophotometer (Hitachi U2001, Tokyo, Japan). Vitamin C was used as a positive control. The percentage of DPPH radical scavenging activity was calculated, and IC_50_ values were determined using GraphPad Prism software (version 5.0, La Jolla, CA, USA).

The ABTS radical scavenging activity was evaluated following Yu et al. (2013) [54]. The ABTS^+^ radical cation was generated by reacting 7 mM ABTS with 2.45 mM potassium persulfate and incubating the mixture in the dark for 12 h at 28 ± 1 °C. The solution was diluted to obtain an absorbance of 0.5–0.6 at 734 nm prior to use. A total of 25 µL of ethanol extract (50–400 µg/mL) was mixed with 1.925 mL of ABTS solution and incubated at 28 °C for 20 min. Absorbance was recorded at 734 nm using a UV–Vis spectrophotometer (Hitachi U2001, Tokyo, Japan). Vitamin C was used as a positive control. The percentage inhibition of ABTS radicals was calculated relative to the blank, and IC_50_ values were determined using GraphPad Prism software (version 5.0, La Jolla, CA, USA).

### 4.5. In Vitro α-Amylase Inhibition Assay

The α-amylase inhibitory activity was determined using the DNSA method of Wickramaratne et al. (2016) [55], with slight modifications. The ethanol extract of *P. schottii* leaves was prepared in phosphate buffer (0.02 M Na_2_HPO_4_/NaH_2_PO_4_, 0.006 M NaCl, pH 6.9) at concentrations of 50–1000 μg/mL. A volume of 200 μL extract was mixed with 200 μL α-amylase (2 U/mL) and incubated at 30 °C for 10 min. Then, 200 μL starch solution (1% *w*/*v*) was added and incubated for 3 min. The reaction was stopped by adding 200 μL DNSA reagent and heating at 85 °C for 10 min. After cooling, 5 mL distilled water was added, and absorbance was measured at 540 nm using a U2001 UV–Vis Spectrophotometer (Hitachi U2001, Tokyo, Japan). Acarbose (Bayer, Berlin, Germany) was used as a positive control. Percentage inhibition was calculated using the standard equation, and IC_50_ values were determined using GraphPad Prism (v5.0).

### 4.6. In Vitro α-Glucosidase Inhibition Assay

The α-glucosidase inhibitory activity was measured using yeast α-glucosidase and pNPG substrate according to Kim et al. (2004) [56]. The reaction mixture contained 100 μL extract (50–800 μg/mL), 50 μL enzyme (1 U/mL), and 250 μL phosphate buffer (0.1 M, pH 6.9). After pre-incubation at 37 °C for 20 min, 10 μL pNPG (10 mM) was added and incubated for 30 min. The reaction was terminated with 650 μL sodium carbonate (1 M), and absorbance was measured at 405 nm using a U2001 UV–Vis Spectrophotometer (Hitachi U2001, Tokyo, Japan). Acarbose (Bayer AG, Berlin, Germany) was employed as a positive control. The inhibition (%) and IC_50_ values were calculated as described above. The α-amylase inhibitory activity assay was performed at 30 °C as recommended in the original protocol [55]. In contrast, the α-glucosidase assay was carried out at 37 °C according to Kim et al. (2004) [56], which is the optimal temperature for yeast α-glucosidase. These temperatures were deliberately maintained to follow the standard and validated conditions for each enzyme as reported in the respective reference methods.

### 4.7. Cytotoxicity and Apoptosis Analysis

The cytotoxic potential of *P. schottii* ethanol leaf extract was evaluated using the MTT assay on human hepatoma HepG2 (ATCC HB-8065) and breast cancer MCF-7 (ATCC HTB-22) cell lines as functional in vitro model systems [57,58]. The cell lines were provided by the Virology Research Laboratory (VRG) group, College of Science, King Saud University, Saudi Arabia. All cell lines were maintained at 37 °C and were mycoplasma-free (LookOut^®^ Mycoplasma qPCR Detection Kit, Merk, Darmstadt, Germany). Cells were cultured in Dulbecco’s Modified Eagle’s Medium (DMEM) supplemented with 10% fetal bovine serum (FBS), 2 mM glutamine, and antibiotic solutions (100 U/mL penicillin and 100 μg/mL streptomycin), and maintained at 37 °C in 5% CO_2_. Cells were seeded in 96-well plates at a density of 1 × 10^4^ cells/well and incubated for 24 h. Subsequently, 100 μL of extract (0–400 μg/mL) was added. Cisplatin (30 μg/mL) was used as a positive control. Following treatment, 10 μL of MTT solution (5 mg/mL) was added and incubated for 2 h in the dark. Formazan crystals were dissolved using 100 μL dimethyl sulfoxide (DMSO), and absorbance was measured at 570 nm using an ELx808 microplate reader (BioTek Laboratories, LLC, Shoreline, WA, USA). IC_50_ values were calculated based on mean absorbance values.

The expression of apoptosis-related genes (caspase-3, caspase-8, caspase-9, Bax, Bcl-2, and Bcl-xL) was analyzed using rRT-PCR following the method described by Aziz et al. (2024) [59]. Cells were seeded at a density of 1 × 10^5^ cells/well and incubated for 24 h. Subsequently, the cells were treated with IC_50_ concentrations of the extract for 48 h. After treatment, cells were trypsinized, centrifuged, and the resulting pellets were collected for RNA extraction. Total RNA was isolated using the RNeasy Micro Kit (Qiagen, Hilden, Germany) according to the manufacturer’s instructions. The extracted RNA was then subjected to rRT-PCR analysis using a 7500 Fast Real-Time PCR System (Applied Biosystems, Foster City, CA, USA). Gene expression analysis was performed following standard rRT-PCR protocols as described in our previous study (Aziz et al. (2024)) [59].

### 4.8. Screening for Antibacterial Activity

#### 4.8.1. Disk Diffusion Method

The antibacterial activity of the ethanol leaf extract of *P. schottii* was evaluated using the disk diffusion method as previously described by Al-Dhabi et al. (2020) [60], with minor modifications. The test microorganisms included Gram-negative bacteria, *E. coli* (ATCC-25922), *K. pneumoniae* (MTCC-13883), and *P. aeruginosa* (MTCC-27853), and Gram-positive strains, *S. aureus* (ATCC-29213), *S. epidermidis* (MTCC 12228), and *E. faecalis* (ATCC-29212). Bacterial suspensions (0.1 mL; 1 × 10^6^ CFU/mL) were evenly spread on Mueller–Hinton agar (MHA) plates and incubated at 37 °C for 24 h. Sterile paper disks were impregnated with 30 μL of extract at concentrations of 50–400 μg/mL and placed onto the inoculated agar surface. Ciprofloxacin (25 μg/mL) was used as a positive control, while disks containing solvent served as negative controls. After incubation at 37 °C for 24 h, the zones of inhibition were measured in millimeters (mm) according to Singh et al. (2016) [61].

#### 4.8.2. Determination of MIC and MBC

The MIC and MBC were determined using the broth microdilution method as described by Basri and Sandra (2016) [62], with slight modifications. Serial dilutions of the ethanol extract (1.95–800 μg/mL) were prepared in Mueller–Hinton broth (MHB) in 96-well microplates. Each well contained 100 μL of MHB and 10 μL of bacterial suspension adjusted to approximately 5 × 10^6^ CFU/mL. The negative control consisted of MHB, while ciprofloxacin (25 μg/mL) was used as a positive control. Plates were incubated at 37 °C for 24 h. Following incubation, 2 mg/mL triphenyl tetrazolium chloride (TTC) solution prepared in phosphate-buffered saline (PBS) was added to each well and incubated for an additional 20 min at 37 °C. Wells showing no color change (colorless) were considered indicative of no bacterial growth, whereas pink coloration indicated bacterial viability. The MIC was defined as having the lowest concentration of extracts that inhibited visible bacterial growth. For MBC determination, aliquots from wells showing no visible growth were sub-cultured onto agar plates and incubated at 37 °C for 24 h. The MBC was defined as the lowest concentration at which no bacterial growth was observed on agar plates, as described by Aljeldah et al. (2022) [63].

### 4.9. Statistical Analysis

All experiments were conducted in triplicate, and data are expressed as the mean ± standard deviation (SD). Statistical comparisons were performed using one-way ANOVA followed by appropriate post hoc tests (e.g., Tukey’s test) using GraphPad Prism (version 5.0, La Jolla, CA, USA). Differences were considered statistically significant at *p* < 0.05.

## 5. Conclusions

The ethanol extract of *P. schottii* leaves contains a high concentration of bioactive phenolics, flavonoids, terpenoids, and fatty acids, all of which exhibit significant in vitro antioxidant, broad-spectrum antibacterial, enzyme inhibitory, and pro-apoptotic cytotoxic effects. These findings strongly imply that this underutilized desert plant has the potential for further development into nutraceuticals or phytopharmaceuticals that target oxidative stress, microbial infections, type 2 diabetes, and cancer. Although the current GC-MS analysis provides relevant data on the lipophilic components of *P. schottii*, the LC–MS/HPLC–MS methods are required to perform a complete analysis of the polar and semi-polar metabolites, including the phenolic and flavonoid metabolites that contributed significantly to TPC and TFC, and to confirm the tentative identification of high-molecular-weight substances. Also, future studies should focus on *in vivo* validation of antidiabetic and anticancer effectiveness in relevant animal models.

## Figures and Tables

**Table 1:** GC-MS analysis of phytochemical compounds identified from the extract of *P. schottii* leaves.

Peak	Rt	Area	Area%	Name	MF	MW	Classification
1	9.06	41,002,083	0.523351	D-Limonene	C_10_H_16_	136	Monoterpenoids
2	10.942	7,201,141	0.091915	2,6-Dimethyl-octa-2,6-dien-1-ol	C_10_H_18_O	154	Monoterpenoids
3	14.535	12,249,870	0.156357	5-Undecene, (Z)-	C_11_H_22_	154	Aliphatic hydrocarbons
4	16.564	13,565,556	0.173151	Linalyl acetate	C_12_H_20_O_2_	196	Monoterpenoids
5	19.796	9,209,444	0.117549	1,5,5-Trimethyl-6-methylene-cyclohexene	C_10_H_16_	136	Monoterpenoids
6	20.057	28,642,023	0.365587	Isochavibetol	C_10_H_12_O_2_	164	Methoxyphenols
7	20.679	8,717,310	0.111268	α-acorenol	C_15_H_26_O	222	Sesquiterpenoids
8	21.332	35,159,513	0.448776	2-Dodecanol	C_12_H_26_O	186	Fatty alcohols
9	22.076	10,166,411	0.129764	Caryophyllene	C_15_H_24_	204	Sesquiterpene
10	22.423	9,565,690	0.122096	Acetic acid, 2,2′-[oxybis(2,1-ethanediyloxy)]bis-	C_8_H_14_O_7_	222	Polyethers/Glycol ether esters
11	24.022	29,738,984	0.379588	β-copaene	C_15_H_24_	204	Sesquiterpenoids
12	25.176	11,631,868	0.148469	α-ylangene	C_15_H_24_	204	Sesquiterpenoids
13	27.508	30,893,664	0.394327	Cetene	C_16_H_32_	224	Aliphatic hydrocarbons
14	28.334	17,818,425	0.227434	Cyclopenta[1,3]cyclopropa[1,2]cyclohepten-3(3aH)-one, 1,2,3b,6,7,8-hexahydro-6,6-dimethyl-	C_13_H_18_O	190	Ketones
15	29.026	21,411,047	0.273291	3-Hydroxy-α-ionene	C_13_H_20_O_2_	208	Sesquiterpenoids
16	29.504	193,725,850	2.472716	Neointermedeol	C_15_H_26_O	222	Sesquiterpenoids
17	30.654	17,838,473	0.22769	trans-Bisabolene epoxide	C_15_H_24_O	220	Sesquiterpenoids
18	30.942	41,595,463	0.530924	5-Thio-D-glucose	C_6_H_12_O_5_S	196	Monosaccharides
19	32.224	17,767,913	0.22679	α-Santonin	C_15_H_18_O_3_	246	Sesquiterpene lactones
20	32.632	63,698,010	0.813041	Tetradecanoic acid	C_14_H_28_O_2_	228	Long-chain fatty acids
21	33.079	22,517,686	0.287416	1-Hexadecanol, 2-methyl-	C_17_H_36_O	256	Long-chain fatty alcohols
22	34.194	96,073,579	1.226283	Neophytadiene	C_20_H_38_	278	Diterpenoids
23	34.819	5,558,213	0.070945	Ethanol, 2-(9-octadecenyloxy)-, (Z)-	C_20_H_40_O_2_	312	Dialkyl ethers
24	35.245	101,663,668	1.297635	Desulphosinigrin	C_10_H_17_NO_6_S	279	Glycosyl compounds
25	36.962	380,021,637	4.850596	Methyl 6-O-[1-methylpropyl]-β-d-galactopyranoside	C_11_H_22_O_6_	250	Glycosyl compounds
26	38.07	1,176,038,831	15.01096	n-Hexadecanoic acid	C_16_H_32_O_2_	256	Long-chain fatty acids
27	40.052	52,433,841	0.669265	Heptadecanoic acid	C_17_H_34_O_2_	270	Long-chain fatty acids
28	40.909	495,156,042	6.320171	Phytol	C_20_H_40_O	296	Acyclic diterpenoids
29	42.132	1,525,900,641	19.47659	Linoleic acid	C_18_H_32_O_2_	280	Linoleic acids and derivatives
30	42.52	106,001,721	1.353006	Octadecanoic acid	C_18_H_36_O_2_	284	Long-chain fatty acids
31	43.406	31,807,085	0.405986	1-Linolenoylglycerol	C_21_H_36_O_4_	352	Linoleic acids and derivatives
32	44.457	56,848,487	0.725614	Arachidonic acid methyl ester	C_21_H_34_O_2_	318	Fatty acid esters
33	45.087	20,065,019	0.25611	Ethyl iso-allocholate	C_26_H_44_O_5_	436	Steroids and steroid derivatives
34	45.867	25,159,444	0.321135	2-Monoolein	C_21_H_40_O_4_	356	Glycerolipids
35	48.133	17,829,776	0.227579	18,19-Secoyohimban-19-oic acid, 16,17,20,21-tetradehydro-16-(hydroxymethyl)-, methyl ester, (15β,16E)-	C_21_H_24_N_2_O_3_	352	Alkaloids
36	48.264	13,460,093	0.171805	8,11,14-Docosatrienoic acid, methyl ester	C_23_H_40_O_2_	348	Fatty acid esters
37	49.476	521,925,111	6.661851	2-Monopalmitin	C_19_H_38_O_4_	330	Glycerolipids
38	51.112	149,619,558	1.909744	25,26,27-Trinorcholecalcifer-24-al	C_24_H_36_O_2_	356	Steroids and steroid derivatives
39	52.902	951,763,500	12.14831	Vitamin E	C_29_H_50_O_2_	430	Tocopherols
40	53.751	326,013,815	4.16124	α-Monostearin	C_21_H_42_O_4_	358	Glycerolipids
41	54.235	401,741,616	5.127829	Cyclopropanebutanoic acid, 2-[[2-[[2-[(2-pentylcyclopropyl)methyl]cyclopropyl]methyl]cyclopropyl]methyl]-, methyl ester	C_25_H_42_O_2_	374	Fatty acid methyl esters
42	55.235	372,951,862	4.760357	Oleamide	C_18_H_35_NO	281	Fatty amides
43	55.991	69,147,443	0.882598	2,2,4-Trimethyl-3-(3,8,12,16-tetramethyl-heptadeca-3,7,11,15-tetraenyl)-cyclohexanol	C_30_H_52_O	428	Triterpenoids
44	56.433	209,608,322	2.67544	Digitoxin	C_41_H_64_O_13_	764	Steroids and steroid derivatives
45	58.002	47,749,603	0.609476	9,10-Secochola-5,7,10(19)-trien-24-al, 3-hydroxy-, (3β,5Z,7E)-	C_24_H_36_O_2_	356	Steroids and steroid derivatives
46	59.111	358,80,279	0.457976	Oleic acid, 3-(octadecyloxy)propyl ester	C_39_H_76_O_3_	592	Fatty acid esters
Total		19.88%		Linoleic acids and derivatives			
		17.85%		Long-chain fatty acids			
		12.15%		Tocopherols			
		11.14%		Glycerolipids			
		6.32%		Acyclic diterpenoids			
		6.15%		Glycosyl compounds			
		5.45%		Steroids and steroid derivatives			
		5.13%		Fatty acid methyl esters			
		4.76%		Fatty amides			
		3.74%		Sesquiterpenoids			

**Table 2:** The inhibitory zone (mm), MIC (μg/mL), and MBC (μg/mL) of *P. schottii leaves* extract.

Bacterium/Dilution	Ciprofloxacin (25 μg/mL)	400 μg/mL	200 μg/mL	100 μg/mL	50 μg/mL	MIC (μg/mL)	MBC (μg/mL)
*S. aureus* (MTCC 29213)	26 ± 1.68	17 ± 2.61	15 ± 0.92	12 ± 0.67	10 ± 0.29	25 ± 0.00	50 ± 0.00
*S. epidermidis* (MTCC 12228)	26 ± 1.55	21 ± 1.98	14 ± 1.36	12 ± 1.95	10 ± 1.75	6.25 ± 0.00	12.50 ± 0.00
*E. faecalis* (ATCC-29212)	27 ± 2.46	20 ± 1.39	17 ± 1.97	13 ± 1.55	11 ± 1.43	6.25 ± 0.00	12.50 ± 0.00
*E. coli* (ATCC 25922)	25 ± 2.17	23 ± 2.55	19 ± 1.26	14 ± 1.48	12 ± 2.34	4.68 ± 2.21	6.25 ± 0.00
*K. pneumoniae* (MTCC 13883)	24 ± 1.35	21 ± 1.46	19 ± 0.91	17 ± 1.13	14 ± 1.74	12.50 ± 0.00	25 ± 0.00
*P. aeruginosa* (MTCC 27853)	23 ± 0.24	23 ± 0.75	21 ± 1.37	18 ± 1.26	15 ± 2.44	9.357 ± 4.14	12.5 ± 0.00

**Note** minimum inhibitory concentration (MIC) and minimum bactericidal concentration (MBC)**.** The reported values are shown in triplicate as the mean ± SD. The results demonstrate a statistically significant decrease from the positive control (25 µg/mL of ciprofloxacin).

## Data Availability

The original contributions presented in this study are included in the article. Further inquiries can be directed to the corresponding author.

## Abbreviations

The following abbreviations are used in this manuscript:

GC–MS	Gas Chromatography–Mass Spectrometry
TPC	Total Phenolic Content
TFC	Total Flavonoid Content
DPPH	2,2-Diphenyl-1-picrylhydrazyl
ABTS	2,2′-Azino-bis(3-ethylbenzothiazoline-6-sulfonic acid)
MIC	Minimum Inhibitory Concentration
MBC	Minimum Bactericidal Concentration
